# Cumulative Incidence of West Nile Virus Infection, Continental United States, 1999–2016

**DOI:** 10.3201/eid2502.180765

**Published:** 2019-02

**Authors:** Shannon E. Ronca, Kristy O. Murray, Melissa S. Nolan

**Affiliations:** Baylor College of Medicine, Houston, Texas, USA (S.E. Ronca, K.O. Murray, M.S. Nolan);; Texas Children’s Hospital, Houston (S.E. Ronca, K.O. Murray, M.S. Nolan)

**Keywords:** West Nile virus, seroprevalence, United States, West Nile neuroinvasive disease, West Nile fever, asymptomatic infections, vector-borne infections, incidence, neuroinvasive disease, ArboNET, viruses, zoonoses

## Abstract

Using reported case data from ArboNET and previous seroprevalence data stratified by age and sex, we conservatively estimate that ≈7 million persons in the United States have been infected with West Nile virus since its introduction in 1999. Our data support the need for public health interventions and improved surveillance.

West Nile virus (WNV) is a mosquito-transmitted flavivirus with human health implications. Since its emergence in 1999, WNV has become endemic across the continental United States (*1*). Seasonal outbreaks occur annually, and large outbreaks occur throughout the country. Infection is commonly asymptomatic; a general febrile illness occurs in ≈20% of the population, and <1% progress to West Nile neuroinvasive disease (WNND), which might include encephalitis, meningitis, and acute flaccid paralysis.

WNV infection can cause permanent sequelae, including physical, neurologic, and cognitive disabilities as well as renal impairment and ocular damage (*2*). The average annual cost to treat hospitalized WNV patients is ≈US $56 million, and initial and long-term costs can exceed US $700,000 per patient (*3*,*4*). Considering the clinical and economic impact of acute and long-term WNV outcomes, determining total WNV disease burden in the United States is imperative. ArboNET data indicate that ≈40% of WNND cases occurred during 2011–2016, suggesting a need to update the estimated cumulative WNV incidence previously determined by Petersen et al. in 2010 (*5*). The objective of our study was to estimate total WNV disease burden in the continental US population during 1999–2016.

## The Study

We collected data from the Centers for Disease Control and Prevention’s ArboNET national surveillance system and performed a comprehensive literature search in PubMed for state-specific and national WNV seroestimates. We used the 2010 US Census database for general population estimates. ArboNET data indicated that the 5 states with the highest clinically reported WNV case counts during 1999–2016 were California (6,504 cases), Texas (5,672 cases), Colorado (5,285 cases), Nebraska (3,911 cases), and South Dakota (2,470 cases) (Appendix Table 3). When evaluating only reported WNND cases, the top 5 states were California (3,390 cases), Texas (3,171 cases), Illinois (1,481 cases), Colorado (1,249 cases), and Louisiana (1,009 cases). The ArboNET dataset demonstrates a cumulative attack rate of 16 cases/100,000 persons in the US population during 1999–2016. When categorizing states into 5 sets by region (Midwest, Northeast, Southeast, Southwest, West), we observed the highest number of cases in the Midwest and West (Figure), a finding that corresponded with the top 5 states of total reported WNV and WNND cases. In the Southwest region, Texas accounts for >55% of the total reported cases.

**Figure:**
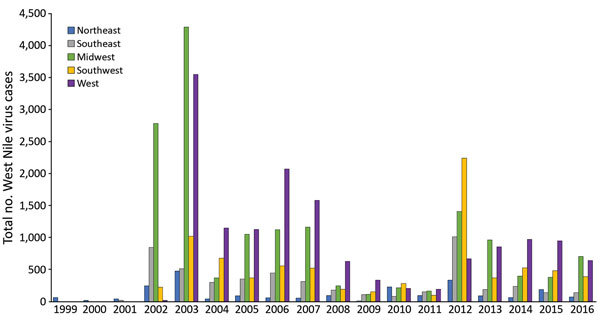
Total West Nile virus cases reported through ArboNET, by year and region, continental United States, 1999–2016.

Next, we estimated cumulative WNV cases for the continental United States using ArboNET-reported WNND cases by state. To determine case estimates among persons >16 years of age, we used Carson et al.’s WNND:infection ratios and 95% CIs stratified by age and sex (*6*). For cases among persons <16 years of age, we applied Mandalakas et al.’s 1:4,200 pediatric WNND:infection ratio and their age ranges for stratified estimates (*7*). We used age groups <15, 15–24, 25–44, 45–64, and >65 years, which is different from the age groups in the original reports (*6*,*7*) because ArboNET reports data by 5-year intervals (e.g., 15–19 years). We used reported ArboNET data for comparison purposes.

Using Carson et al.’s estimates of seroprevalence in adults and Mandalakas et al.’s estimates in children (*6*,*7*) to stratify by age and sex, we estimate that ≈7 (95% CI 5.7–8.1) million WNV infections have occurred in the United States since WNV was introduced (Appendix Tables 1, 2). This number equates to ≈2.2% of the US population, greater than the estimate for 1999–2010 reported by Petersen et al. (1.1% of the population, 3 million infections) (*5*) and ArboNET (0.16% of the population).

Since Petersen et al.’s previous estimate (*5*), 40% of all WNND cases have been reported. Our estimate of infections occurring during 1999–2016 is generally consistent with the incremental infection burden for the last 6 years of our study period. Disease burden estimates might be affected by the changing epidemiology or disease penetration over the past 17 years. For instance, the ratio of neuroinvasive to nonneuroinvasive cases varies by geographic locality and is likely related to differences in testing, surveillance, and access to care (*8*,*9*). Furthermore, infection trends might vary during each major epidemic. In 2003, the Midwest states of Nebraska and Colorado had the highest incidence rates (*10*), but in 2012, Texas had the highest (*9*). However, a study looking at blood donors indicates that WNND:infection ratios have not changed over time (*11*) and an additional study has confirmed the accuracy of Carson et al.’s estimates (*6*,*12*). This information highlights the need for national standards for localized surveillance and reporting for more accurate estimates of disease burden and predictions of future disease severity.

In reality, the number of infections is likely higher than what was calculated here, as underdiagnosis is evident; a study by Vanichanan et al. indicated that patients are tested for WNV infection only one third of the time when viral encephalitis is clinically diagnosed (*8*). Increased awareness in the medical community will be needed not only for proper diagnosis of cases but also for quick implementation of control measures to prevent further cases and the improvement of surveillance data.

When evaluating disease burden, we must discuss how vulnerable, high-risk populations, such as those who are homeless, affect estimates. Only 1 study explicitly defines the relationship between WNV and homelessness (*13*). In that study, 6.8% of homeless persons in Houston, Texas, were seropositive for WNV infection after only 2 transmission seasons, and seroprevalence was even higher (17%) when specifically evaluating those who slept outdoors. According to the US Department of Housing and Urban Development, nearly 550,000 of the US population were homeless on any given day in 2016; ≈32% of these persons lived in unsheltered conditions, and ≈14% were considered chronically homeless (https://www.hud.gov). Because the burden of disease among homeless persons is difficult to delineate without additional studies, this unique population was not included in our estimate.

Our study has a few other notable limitations. Census data are not an exact representation of the population but an estimate of the number of persons at a given time. We also cannot account for cases in which persons do not seek treatment. Despite these limitations, our updated estimate helps to provide data for future economic burden estimates and cost-effectiveness studies for vaccines and novel therapeutics. A WNV vaccine was previously thought to not be cost-effective (*14*), but a study published in 2017 indicated an age-targeted vaccination program would improve cost-effectiveness (*15*). Cost-effectiveness data and our new estimates of infection demonstrate that a high proportion of the population is seronegative and still susceptible to WNV infection, providing additional support that region-targeted vaccinations could be beneficial to the US population and should be further explored.

## Conclusions

We estimate that ≈7 (95% CI 5.7–8.1) million persons in the continental United States were infected with WNV during 1999–2016, more than double the 2010 estimate of 3 million infections. Our estimate highlights the need for improved disease surveillance and reporting. As the cumulative incidence continues to climb, our findings provide additional support for the economic benefit of insecticide and vaccine interventions, especially in the Midwest, Southwest, and West of the United States; nearly 98% of the US population remains vulnerable to WNV infection.

Appendix Seroprevalence or cumulative incidence estimates of West Nile virus, by age, sex, and state, continental United States, 1999–2016
