# Supplementary material for: Cumulative Incidence of West Nile Virus Infection, Continental United States, 1999–2016
Source: Emerg Infect Dis. 2019 Feb;25(2):325–7. doi: 10.3201/eid2502.180765 (PMC6346444; doi:10.3201/eid2502.180765)
Supplement: Appendix — Seroprevalence or cumulative incidence estimates of West Nile virus, by age, sex, and state, continental United States, 1999–2016 [file 18-0765-Techapp-s1.pdf]

Article DOI: https://doi.org/10.3201/eid2502.180765

Cumulative Incidence of West Nile Virus Infection, Continental United States, 1999–2016

Appendix

Appendix Table 1. Estimated West Nile virus seroprevalence, by state, sex, and age, continental United States, 1999–2016*

	Male population, age, y, no. seropositive					Female population, age, y, no. seropositive					
State	<15	15–24	25–44	45–64	≥65	<15	15–24	25–44	45–64	≥65	Estimated total (95% CI)
Alabama	4,201	1,440	6,069	11,703	2,244	4,201	–	2,317	7,760	1,922	41,857 (34,958–50,876)
Arizona	54,613	14,400	42,483	27,639	10,098	37,809	16,016	24,163	53,544	7,936	288,701 (247,336–344,338)
Arkansas	16,804	6,480	6,783	10,956	2,703	12,603	4,928	7,613	12,804	2,170	83,844 (71,960–99,103)
California	147,035	61,920	110,670	211,650	34,680	84,020	55,440	55,608	158,692	25,358	945,073 (796,377–1,135,877)
Colorado	46,211	25,920	54,621	58,266	11,475	42,010	43,120	48,326	72,168	9,548	411,665 (340,679–501,771)
Connecticut	4,201	1,440	1,428	5,478	1,020	–	1,232	2,317	3,880	1,116	22,112 (18,118–27,152)
Delaware	4,201	720	–	249	408	–	2,464	–	1,552	186	9,780 (8,439–11,518)
DC	–	–	1,071	2,490	867	4,201	–	1,655	3,104	496	13,884 (12,034–16,560)
Florida	21,005	6,480	12,852	18,177	2,703	12,603	–	6,951	14,744	1,674	97,189 (84,646–113,625)
Georgia	21,005	5,760	6,069	14,691	2,295	4,201	2,464	4,303	7,760	2,356	70,904 (60,473–83,449)
Idaho	33,608	4,320	6,069	9,711	1,836	12,603	3,696	7,613	13,192	1,674	94,322 (84,589–107,494)
Illinois	54,613	23,760	42,483	59,511	15,555	37,809	34,496	41,706	101,268	15,500	426,701 (358,944–521,001)
Indiana	21,005	8,640	9,996	16,434	3,876	21,005	8,624	12,578	24,444	3,782	130,384 (111,964–155,196)
Iowa	12,603	10,080	10,353	11,205	2,499	16,804	4,928	6,289	10,476	1,984	87,221 (738,68–103,366)
Kansas	33,608	2,880	10,710	15,438	3,672	8,402	11,088	7,613	14,356	2,480	110,247 (94,921–129,445)
Kentucky	4,201	–	2,856	2,988	2,040	4,201	1,232	5,296	3,492	1,116	27,422 (22,628–33,223)
Louisiana	67,216	20,160	39,627	45,816	10,710	50,412	38,192	28,797	53,544	9,424	363,898 (308,216–433,888)
Maine	–	–	357	249	–	–	–	–	–	–	606 (467–749)
Maryland	4,201	4,320	4,284	10,956	2,805	–	2,464	3,972	14,744	2,666	50,412 (41,364–63,318)
Massachusetts	8,402	1,440	1,785	5,229	1,020	–	–	1,655	7,760	1,488	28,779 (25,162–34,421)
Michigan	50,412	11,520	31,059	49,800	11,475	21,005	17,248	31,114	52,380	9,920	285,933 (239,959–345,932)
Minnesota	21,005	4,320	12,852	12,201	3,723	8,402	9,856	6,289	11,640	1,488	91,776 (77,489–109,223)
Mississippi	63,015	14,400	27,489	31,872	7,803	33,608	23,408	14,564	34,532	7,130	257,821 (221,077–303,858)
Missouri	25,206	6,480	13,566	23,904	5,049	4,201	9,856	11,254	23,280	4,278	127,074 (106,130–154,224)
Montana	12,603	2,880	4,641	6,972	1,683	8,402	2,464	5,958	12,416	1,178	59,197 (52,051–69,553)
Nebraska	54,613	15,120	27,489	29,631	6,375	25,206	18,480	23,832	33,756	3,968	238,470 (203,623–282,255)
Nevada	4,201	720	2,856	5,478	2,091	4,201	2,464	2,317	8,924	1,178	34,430 (29,494–41,707)
New Hampshire	–	720	714	249	51	–	–	–	–	–	1,734 (1,273–2,198)
New Jersey	–	720	5,712	6,723	2,397	–	–	4,303	6,208	1,736	27,799 (21,554–35,782)
New Mexico	25,206	5,040	8,211	14,442	3,978	16,804	11,088	7,944	14,356	2,294	109,363 (94,165–128,385)
New York	25,206	5,760	15,708	28,635	9,231	8,402	7,392	9,930	28,712	6,634	145,610 (121,475–177,515)
North Carolina	4,201	–	2,499	1,992	510	4,201	1,232	1,655	2,716	248	19,254 (17,012–22,224)

State	Male population, age, y, no. seropositive					Female population, age, y, no. seropositive					Estimated total (95% CI)
	<15	15–24	25–44	45–64	≥65	<15	15–24	25–44	45–64	≥65	
North Dakota	16,804	11,520	12,138	15,936	4,029	8,402	9,856	8,937	15,132	2,294	105,048 (86,790–127,315)
Ohio	25,206	5,760	25,347	25,896	9,690	33,608	13,552	20,853	27,936	7,750	195,598 (163,128–235,855)
Oklahoma	21,005	7,920	11,781	23,655	6,069	12,603	9,856	9,599	16,296	5,022	123,806 (102,379–149,730)
Oregon	–	720	–	1,743	306	–	2,464	1,324	2,328	372	9,257 (7,178–11,936)
Pennsylvania	8,402	7,920	6,426	14,442	3,876	25,206	14,784	9,599	21,728	3,720	116,103 (97,991–139,894)
Rhode Island	–	720	357	249	102	–	–	–	1,940	124	3,492 (3,057–4,413)
South Carolina	–	720	1,785	2,490	510	–	1,232	662	3,104	372	10,875 (8,737–13,826)
South Dakota	21,005	12,960	26,775	18,426	4,590	54,613	14,784	17,874	26,772	3,038	200,837 (173,049–235,744)
Tennessee	4,201	4,320	6,426	8,466	2,703	12,603	3,696	3,641	8,148	3,348	57,552 (47,494–69,891)
Texas	201,648	66,240	110,313	163,842	35,241	92,422	76,384	79,440	144,724	25,978	996,232 (839,980–1,191,226)
Utah	16,804	3,600	3,927	8,466	1,071	12,603	9,856	5,627	5,044	992	67,990 (58,388–78,948)
Vermont	–	–	–	498	51	–	–	–	–	–	549 (439–660)
Virginia	12,603	–	3,213	6,225	1,836	–	–	1,655	4,268	930	30,730 (26,977–35,661)
Washington	4,201	720	2,142	2,739	714	–	–	662	2,328	434	13,940 (11,937–16,574)
West Virginia	–	–	357	996	306	–	–	–	388	124	2,171 (1,697–2,757)
Wisconsin	12,603	2,160	4,641	8,466	1,785	8,402	3,696	1,986	6,208	1,302	51,249 (44,547–59,628)
Wyoming	25,206	2,160	3,927	10,458	1,377	4,201	11,088	6,289	10,088	1,426	76,220 (65,740–89,346)
Total (95% CI)	1,214,089 (–)	395,280 (281,088–506,178)	742,917 (547,303–946,855)	1,063,728 (871,488–1,255,968)	241,128 (146,568–340,416)	751,979 (–)	505,120 (335,380–675,270)	556,080 (391,440–722,400)	1,104,636 (1,127,412–1,355,172)	190,154 (55,206–334,303)	6,765,111 (5,721,953–8,102,630)

*Case estimates were calculated by using ArboNET data for number of WNND cases by state and WNND:infection ratios reported in previous studies. For persons ≥16 years of age, the age- and sex-stratified WNND:infection estimates and 95% CIs from Carson et al. (*1*) were used. For persons <16 years of age, the WNND:infection ratio of 1:4,200 (1 case reported for every 4,200 undetected cases) calculated by Mandalakas et al. (*2*) was used; 95% CIs were not reported in this study and were not calculated for this age group in this study. All 95% CIs calculated can be found in Appendix Table 2. WNND, West Nile neuroinvasive disease.

Appendix Table 2. Estimated 95% CIs of West Nile virus seroprevalence, by state, sex, and age, continental United States, 1999–2016*

	Male population, age, y, 95% CI of seroprevalence				Female population, age, y, 95% CI of seroprevalence				
State	15–24	25–44	45–64	>65	15–24	25–44	45–64	>65	Total
Alabama	1,024–1,844	4,471–7,735	9,588–13,818	1,364–3,168	–	1,631–3,010	7,920–9,520	558–3,379	34,958–50,876
Arizona	10,240–18,440	31,297–54,145	22,644–32,634	6,138–14,256	10,634–21,411	17,009–31,390	54,648–65,688	2,304–13,952	247,336–344,338
Arkansas	4,608–8,298	4,997–8,645	8,976–12,936	1,643–3,816	3,272–6,588	5,359–9,890	13,068–15,708	630–3,815	71,960–99,103
California	44,032–79,292	81,530–141,050	173,400–249,900	21,080–48,960	36,810–74,115	39,144–72,240	161,964–194,684	7,362–4,4581	796,377–1,135,877
Colorado	18,432–33,192	40,239–69,615	47,736–68,796	6,975–16,200	28,630–57,645	34,018–62,780	73,656–88,536	2,772–16,786	340,679–501,771
Connecticut	1,024–1,844	1,052–1,820	4,488–6,468	620–1,440	818–1,647	1,631–3,010	3,960–4,760	324–1,962	18,118–27,152
Delaware	512–922	–	204–294	248–576	1,636–3,294	–	1,584–1,904	54–327	8,439–11,518
DC	–	789–1,365	2,040–2,940	527–1,224	–	1,165–2,150	3,168–3,808	144–872	12,034–16,560
Florida	4,608–8,298	9,468–16,380	14,892–21,462	1,643–3,816	–	4,893–9,030	15,048–18,088	486–2,943	84,646–113,625
Georgia	4,096–7,376	4,471–7,735	12,036–17,346	1,395–3,240	1,636–3,294	3,029–5,590	7,920–9,520	684–4,142	60,473–83,449
Idaho	3,072–5,532	4,471–7,735	7,956–11,466	1,116–2,592	2,454–4,941	5,359–9,890	13,464–16,184	486–2,943	84,589–107,494
Illinois	16,896–30,426	31,297–54,145	48,756–70,266	9,455–21,960	22,904–46,116	29,358–54,180	103,356–124,236	4,500–27,250	358,944–521,001
Indiana	6,144–11,064	7,364–12,740	13,464–19,404	2,356–5,472	5,726–11,529	8,854–16,340	24,948–29,988	1,098–6,649	111,964–155,196
Iowa	7,168–12,908	7,627–13,195	9,180–13,230	1,519–3,528	3,272–6,588	4,427–8,170	10,692–12,852	576–3,488	73,868–103,366
Kansas	2,048–3,688	7,890–13,650	12,648–18,228	2,232–5,184	7,362–14,823	5,359–9,890	14,652–17,612	720–4,360	94,921–129,445
Kentucky	–	2,104–3,640	2,448–3,528	1,240–2,880	818–1,647	3,728–6,880	3,564–4,284	324–1,962	22,628–33,223
Louisiana	14,336–25,816	29,193–50,505	37,536–54,096	6,510–15,120	25,358–51,057	20,271–37,410	54,648–65,688	2,736–16,568	308,216–433,888
Maine	–	263–455	204–294	–	–	–	–	–	467–749
Maryland	3,072–5,532	3,156–5,460	8,976–12,936	1,705–3,960	1,636–3,294	2,796–5,160	15,048–18,088	774–4,687	41,364–63,318
Massachusetts	1,024–1,844	1,315–2,275	4,284–6,174	620–1,440	–	1,165–2,150	7,920–9,520	432–2,616	25,162–34,421
Michigan	8,192–14,752	22,881–39,585	40,800–58,800	6,975–16,200	11,452–23,058	21,902–40,420	53,460–64,260	2,880–17,440	239,959–345,932
Minnesota	3,072–5,532	9,468–16,380	9,996–14,406	2,263–5,256	6,544–13,176	4,427–8,170	11,880–14,280	432–2,616	77,489–109,223
Mississippi	10,240–18,440	20,251–35,035	26,112–37,632	4,743–11,016	15,542–31,293	10,252–18,920	35,244–42,364	2,070–12,535	221,077–303,858
Missouri	4,608–8,298	9,994–17,290	19,584–28,224	3,069–7,128	6,544–13,176	7,922–14,620	23,760–28,560	1,242–7,521	106,130–154,224
Montana	2,048–3,688	3,419–5,915	5,712–8,232	1,023–2,376	1,636–3,294	4,194–7,740	12,672–15,232	342–2,071	52,051–69,553
Nebraska	10,752–19,362	20,251–35,035	24,276–34,986	3,875–9,000	12,270–24,705	16,776–30,960	34,452–41,412	1,152–6,976	203,623–282,255
Nevada	512–922	2,104–3,640	4,488–6,468	1,271–2,952	1,636–3,294	1,631–3,010	9,108–10,948	342–2,071	29,494–41,707
New Hampshire	512–922	526–910	204–294	31–72	–	–	–	–	1,273–2,198
New Jersey	512–922	4,208–7,280	5,508–7,938	1,457–3,384	–	3,029–5,590	6,336–7,616	504–3,052	21,554–35,782
New Mexico	3,584–6,454	6,049–10,465	11,832–17,052	2,418–5,616	7,362–14,823	5,592–10,320	14,652–17,612	666–4,033	94,165–128,385
New York	4,096–7,376	11,572–20,020	23,460–33,810	5,611–13,032	4,908–9,882	6,990–12,900	29,304–35,224	1,926–11,663	121,475–177,515
North Carolina	–	1,841–3,185	1,632–2,352	310–720	818–1,647	1,165–2,150	2,772–3,332	72–436	17,012–22,224
North Dakota	8,192–14,752	8,942–15,470	13,056–18,816	2,449–5,688	6,544–13,176	6,291–11,610	15,444–18,564	666–4,033	86,790–127,315
Ohio	4,096–7,376	18,673–32,305	21,216–30,576	5,890–13,680	8,998–18,117	14,679–27,090	28,512–34,272	2,250–13,625	163,128–235,855
Oklahoma	56,32–10,142	8,679–15,015	19,380–27,930	3,689–8,568	6,544–13,176	6,757–12,470	16,632–19,992	1,458–8,829	102,379–149,730
Oregon	512–922	–	1,428–2,058	186–432	1,636–3,294	932–1,720	2,376–2,856	108–654	7,178–11,936
Pennsylvania	5,632–10,142	4,734–8,190	11,832–17,052	2,356–5,472	9,816–19,764	6,757–12,470	22,176–26,656	1,080–6,540	97,991–139,894
Rhode Island	512–922	263–455	204–294	62–144	–	–	1,980–2,380	36–218	3,057–4,413
South Carolina	512–922	1,315–2,275	2,040–2,940	310–720	818–1,647	466–860	3,168–3,808	108–654	8,737–13,826
South Dakota	9,216–16,596	19,725–34,125	15,096–21,756	2,790–6,480	9,816–19,764	12,582–23,220	27,324–32,844	882–5,341	173,049–235,744
Tennessee	3,072–5,532	4,734–8,190	6,936–9,996	1,643–3,816	2,454–4,941	2,563–4,730	8,316–9,996	972–5,886	47,494–69,891
Texas	47,104–84,824	81,267–140,595	134,232–193,452	21,421–49,752	50,716–102,114	55,920–103,200	147,708–177,548	7,542–45,671	839,980–1,191,226
Utah	2,560–4,610	2,893–5,005	6,936–9,996	651–1,512	6,544–13,176	3,961–7,310	5,148–6,188	288–1,744	58,388–78,948

State	Male population, age, y, 95% CI of seroprevalence				Female population, age, y, 95% CI of seroprevalence				Total
	15–24	25–44	45–64	≥65	15–24	25–44	45–64	≥65	
Vermont	–	–	408–588	31–72	–	–	–	–	439–660
Virginia	–	2,367–4,095	5,100–7,350	1,116–2,592	–	1,165–2,150	4,356–5,236	270–1,635	26,977–35,661
Washington	512–922	1,578–2,730	2,244–3,234	434–1,008	–	466–860	2,376–2,856	126–763	11,937–16,574
West Virginia	–	263–455	816–1,176	186–432	–	–	396–476	36–218	1,697–2,757
Wisconsin	1,536–2,766	3,419–5,915	6,936–9,996	1,085–2,520	2,454–4,941	1,398–2,580	6,336–7,616	378–2,289	44,547–59,628
Wyoming	1,536–2,766	2,893–5,005	8,568–12,348	837–1,944	7,362–14,823	4,427–8,170	10,296–12,376	414–2,507	65,740–89,346
Total	281,088–506,178	547,303–946,855	871,488–1,255,968	146,568–340,416	335,380–675,270	391,440–722,400	1,127,412–1,355,172	55,206–334,303	572,195–8,102,630

*Calculated by using age- and sex-stratified WNND:infection estimates and 95% CIs from Carson et al. (*1*). WNND, West Nile neuroinvasive disease.

Appendix Table 3. National and state cumulative incidence estimates of reported WNV infection, continental United States, 1999–2016*

State	Region	2010 Census state population	ArboNET data: WNND cases	Nonneuroinvasive cases	Presumptive viremic blood donors	Total no. recorded WNV infections	Cumulative attack rate per 100,000 population	Estimated total infections†	Estimated seroprevalence (95% CI), %
Alabama	Southeast	4,779,736	183	83	21	287	6	41,857	0.9 (0.7–1.1)
Arizona	Southwest	6,392,017	963	551	214	1,728	27	288,701	4.5 (3.9–5.4)
Arkansas	Southeast	2,915,918	235	76	12	323	11	83,844	2.9 (2.5–3.4)
California	West	37,253,956	3,390	2,446	668	6,504	17	945,073	2.5 (2.1–3.0)
Colorado	West	5,029,196	1,249	3,834	202	5,285	105	411,665	8.2 (6.8–10.0)
Connecticut	Northeast	3,574,097	86	43	10	139	4	22,112	0.6 (0.5–0.8)
Delaware	Northeast	897,934	20	21	5	46	5	9,780	1.1 (0.9–1.3)
Florida	Southeast	18,801,310	271	90	26	387	2	97,189	0.5 (0.5–0.6)
Georgia	Southeast	9,687,653	213	169	60	442	5	70,904	0.7 (0.6–0.9)
Idaho	West	1,567,582	201	266	61	528	34	94,322	6.0 (5.4–6.9)
Illinois	Midwest	12,830,632	1,481	868	94	2,443	19	426,701	3.3 (2.8–4.1)
Indiana	Midwest	6,483,802	378	228	65	671	10	130,384	2.0 (1.7–2.4)
Iowa	Midwest	3,046,355	242	243	72	557	18	87,221	2.9 (2.4–3.4)
Kansas	Midwest	2,853,118	305	272	343	920	32	110,247	3.9 (3.3–4.5)
Kentucky	Southeast	4,339,367	111	50	36	197	5	27,422	0.6 (0.5–0.8)
Louisiana	Southeast	4,533,372	1,009	586	161	1,756	39	363,898	8.0 (6.8–9.6)
Maine	Northeast	1,328,361	2	0	0	2	0	606	0 (0–0.1)
Maryland	Northeast	5,773,552	219	76	46	341	6	50,412	0.9 (0.7–1.1)
Massachusetts	Northeast	6,547,629	109	35	11	155	2	28,779	0.4 (0.4–0.5)
Michigan	Midwest	9,883,640	990	162	68	1,220	12	285,933	2.9 (2.4–3.5)
Minnesota	Midwest	5,303,925	290	393	132	815	15	91,776	1.7 (1.5–2.1)
Mississippi	Southeast	2,967,297	700	519	84	1,303	44	257,821	8.7 (7.5–10.2)
Missouri	Midwest	5,988,927	429	90	56	575	10	127,074	2.1 (1.8–2.6)
Montana	West	989,415	158	380	20	558	56	59,197	6.0 (5.3–7.0)
Nebraska	Midwest	1,826,341	639	2,795	477	3,911	214	238,470	13.1 (11.1–15.5)
Nevada	West	2,700,551	138	77	49	264	10	34,430	1.3 (1.1–1.5)
New Hampshire	Northeast	1,316,470	5	1	0	6	0	1,734	0.1 (0.1–0.2)
New Jersey	Northeast	8,791,894	159	74	21	254	3	27,799	0.3 (0.2–0.4)
New Mexico	Southwest	2,059,179	289	278	55	622	30	109,363	5.3 (4.6–6.2)
New York	Northeast	19,378,102	595	205	67	867	4	145,610	0.8 (0.6–0.9)
North Carolina	Southeast	9,535,483	48	15	6	69	1	19,254	0.2 (0.2–0.2)
North Dakota	Midwest	672,591	334	1,184	121	1,639	244	105,048	15.6 (12.9–18.9)
Ohio	Midwest	11,536,504	670	262	80	1,012	9	195,598	1.7 (1.4–2.0)
Oklahoma	Southwest	3,751,351	447	281	155	883	24	123,806	3.3 (2.7–4.0)
Oregon	West	381,074	35	74	11	120	31	9,257	2.4 (1.9–3.1)
Pennsylvania	Northeast	12,702,379	342	197	24	563	4	116,103	0.9 (0.8–1.1)
Rhode Island	Northeast	1,052,567	14	2	0	16	2	3,492	0.3 (0.3–0.4)
South Carolina	Southeast	4,625,364	49	24	22	95	2	10,875	0.2 (0.2–0.3)
South Dakota	Midwest	814,180	494	1,790	186	2,470	303	200,837	24.7 (21.3–29.0)
Tennessee	Southeast	6,346,105	207	71	12	290	5	57,552	0.9 (0.7–1.1)
Texas	Southwest	25,145,561	3,171	1,980	521	5,672	23	996,232	4.0 (3.3–4.7)
Utah	West	3,763,885	140	118	44	302	8	67,990	1.8 (1.6–2.1)
Vermont	Northeast	625,741	5	8	1	14	2	549	0.1 (0.1–0.1)
Virginia	Southeast	8,001,024	110	48	29	187	2	30,730	0.4 (0.3–0.4)
Washington	West	6,724,540	56	43	26	125	2	13,940	0.2 (0.2–0.2)
West Virginia	Southeast	1,852,994	14	13	4	31	2	2,171	0.1 (0.1–0.1)

State	Region	2010 Census state population	ArboNET data					Estimated total infections†	Estimated seroprevalence (95% CI), %
			WNND cases	Nonneuroinvasive cases	Presumptive viremic blood donors	Total no. recorded WNV infections	Cumulative attack rate per 100,000 population		
Wisconsin	Midwest	5,686,986	150	87	177	414	7	51,249	0.9 (0.8–1.0)
Wyoming	West	563,626	175	515	31	721	128	76,220	13.5 (11.7–15.9)
Total		303,623,283	21,520	21,623	4,586	47,729	16	6,751,227	2.2 (1.9–2.7)

*WNND, West Nile neuroinvasive disease; WNV, West Nile virus.
†Estimated total from Appendix Table 1.

References

1. Carson PJ, Borchardt SM, Custer B, Prince HE, Dunn-Williams J, Winkelman V, et al. Neuroinvasive disease and West Nile virus infection, North Dakota, USA, 1999–2008. Emerg Infect Dis. 2012;18:684–6. PubMed http://dx.doi.org/10.3201/eid1804.111313

2. Mandalakas AM, Kippes C, Sedransk J, Kile JR, Garg A, McLeod J, et al. West Nile virus epidemic, northeast Ohio, 2002. Emerg Infect Dis. 2005;11:1774–7. PubMed http://dx.doi.org/10.3201/eid1111.040933
